# Ephrin-B1 Is a Novel Biomarker of Bladder Cancer Aggressiveness. Studies in Murine Models and in Human Samples

**DOI:** 10.3389/fonc.2020.00283

**Published:** 2020-03-27

**Authors:** María Victoria Mencucci, Lara Lapyckyj, Marina Rosso, María José Besso, Denise Belgorosky, Mariana Isola, Silvia Vanzulli, Catalina Lodillinsky, Ana María Eiján, Juan Carlos Tejerizo, Matías Ignacio Gonzalez, María Ercilia Zubieta, Mónica Hebe Vazquez-Levin

**Affiliations:** ^1^Laboratorio de Estudios de la Interacción Celular en Reproducción y Cáncer, Instituto de Biología y Medicina Experimental (IBYME; CONICET-FIBYME), Buenos Aires, Argentina; ^2^Research Area, Instituto de Oncología Angel H. Roffo, Universidad de Buenos Aires, Buenos Aires, Argentina; ^3^Departamento de Anatomía Patológica, Hospital Italiano de Buenos Aires, Buenos Aires, Argentina; ^4^Academia Nacional de Medicina, Buenos Aires, Argentina; ^5^Departamento de Urología, Hospital Italiano de Buenos Aires, Buenos Aires, Argentina

**Keywords:** bladder cancer, epithelial cadherin, beta-catenin, Ephrin-B1, biomarker

## Abstract

Bladder cancer (BC) is the ninth most common cancer worldwide, but molecular changes are still under study. During tumor progression, Epithelial cadherin (E-cadherin) expression is altered and β-catenin may be translocated to the nucleus, where it acts as co-transcription factor of tumor invasion associated genes. This investigation further characterizes E-cadherin and β-catenin associated changes in BC, by combining bioinformatics, an experimental murine cell model (MB49/MB49-I) and human BC samples. In *in silico* studies, a DisGeNET (gene-disease associations database) analysis identified *CDH1* (E-cadherin gene) as one with highest score among 130 BC related-genes. COSMIC mutation analysis revealed *CDH1* low mutations rates. Compared to MB49 control BC cells, MB49-I invasive cells showed decreased E-cadherin expression, E- to P-cadherin switch, higher β-catenin nuclear signal and lower cytoplasmic p-Ser33-β-catenin signal, higher Ephrin-B1 ligand and EphB2 receptor expression, higher Phospho-Stat3 and Urokinase-type Plasminogen Activator (UPA), and UPA receptor expression. MB49-I cells transfected with Ephrin-B1 siRNA showed lower migratory and invasive capacity than control cells (scramble siRNA). By immunohistochemistry, orthotopic MB49-I tumors had lower E-cadherin, increased nuclear β-catenin, lower pSer33-β-catenin cytoplasmic signal, and higher Ephrin-B1 expression than MB49 tumors. Similar changes were found in human BC tumors, and 83% of infiltrating tumors depicted a high Ephrin-B1 stain. An association between higher Ephrin-B1 expression and higher stage and tumor grade was found. No association was found between abnormal E-cadherin signal, Ephrin-B1 expression or clinical-pathological parameter. This study thoroughly analyzed E-cadherin and associated changes in BC, and reports Ephrin-B1 as a new marker of tumor aggressiveness.

## Introduction

Bladder cancer (BC) is one of the ten solid tumors with highest incidence and mortality worldwide, with 549,393 new cases and 199,922 deaths reported in 2018, and with an increase of 50% expected for 2040 (International Agency for Research on Cancer; WHO^1^). Clinically, it is classified as non-muscle-invasive (NMIBC) and muscle-invasive (MIBC) or infiltrating BC, being the latter highly aggressive. Urothelial tumors represent >90% of the cases; while 75% are limited to the mucosa or submucosa at the time of diagnosis, 10–30% NMIBC patients progress to MIBC and 50–70% have tumor recurrence (1). Thus, muscle invasion is a key event in BC, being important to identify MIBC biomarkers for accurate treatment and understanding the underlying mechanisms to prevent tumor progression and aggressiveness.

Among molecular changes associated to BC, a decreased expression of epithelial cadherin (E-cadherin) has long been reported (2). In a systematic review and meta-analysis, reduced E-cadherin was associated with poor BC prognosis (3); however, the underlying mechanisms have not been fully characterized. E-cadherin is the founder member of the cadherin superfamily (4). It is a 120 kDa transmembrane glycoprotein encoded by the *CDH1* gene; its extracellular domain mediates cell-cell adhesion, while the cytoplasmic domain binds to β-catenin that links E-cadherin to the actin cytoskeleton, and is involved in signal transduction (5). E-cadherin decrease/loss expression is a hallmark of Epithelial-to-Mesenchymal Transition (EMT) that promotes cell motility/invasive behavior, cancer progression and metastasis (6, 7). Alterations in E-cadherin expression during EMT are accompanied by increased expression of transcriptional represors, β-catenin loss at the cell membrane, and filamentous actin (F-actin) belt replacement by a network of stress fibers. Also, it is tipically characterized by an increased expression of neural (N-cadherin) and, in some cases, by placental (P-cadherin) cadherin, a phenomenon called cadherin switch. Some evidence of EMT-related events has been reported in BC (8–10).

This report further characterizes alterations in E-cadherin expression and EMT-related events in BC with the aim to identify novel markers of BC progression. Studies were addressed in the MB49 and MB49-I murine model of tumor progression (11, 12), and in BC patient tissue samples.

## Materials

Chemicals were of analytical and tissue culture grade and purchased from BioRad (Richmond, CA, USA), Thermo-Fisher Scientific (Carlsbad, CA, USA), and Sigma Chemical Co. (St. Louis, MO, USA), unless specifically indicated. Primary antibodies used were: Anti-E-cadherin: (1) 610181 (BD Biosciences, San Diego, CA, USA), (2) HECD-1 (Thermo); Anti-β-catenin (610153; BD); Anti-phospho-Ser33-β-catenin (pSer33-β-catenin; Ser33-R; SCB); Anti-N-cadherin (H-63, SCB); Anti-P-cadherin (H-105, SCB); Anti-Ephrin-B1 (A-20, SCB); Anti-EphB2 (H-80, SCB); Anti-Signal transducer and activator of transcription 3 (STAT3) (B-7, SCB); Anti-phospho-STAT3 (pSTAT3) (C-20, SCB); Anti-Proliferating cell nuclear antigen (PCNA) (PC10, SCB), Anti-actin (I-19, SCB); Anti-β-tubulin (D-66, Sigma). Secondary antibodies used were Cy3-labeled anti-mouse or anti-rabbit (Sigma) and FITC-labeled anti-mouse (Vector Lab. Inc., Burlingame, CA, USA) IgGs for fluorescence immunocytochemistry, Anti-mouse (Vector) or Anti-rabbit (Sigma) IgGs coupled to horseradish peroxidase for Western immunoblotting. In control experiments, primary antibodies were replaced by purified mouse or rabbit IgGs, as required.

### Murine Cell Lines and Tumors

Established MB49 and MB49-I mouse cell lines were used as experimental models. The MB49 cell line was generated from an *in vitro* neoplastic transformation of mouse bladder epithelium primary cultures (13). The MB49-I cell line was originated after *in vivo* successive passages of a primary tumor obtained by subcutaneous inoculation of MB49 cells in C57Bl/6J males (11).

Murine bladder tumors were generated by orthotopic inoculation of MB49 and MB49-I cells into C57BL/6 mice bladder (11). Mice were handled in accordance with the international procedure for Care and Use of Laboratory Animals; a protocol was approved by the Institute of Oncology Angel H. Roffo Review Board (**#**2012/02).

### Human Tumor Samples

Human BC tissue samples were obtained from patients diagnosed with urothelial BC at Hospital Italiano of Buenos Aires, between 2012 and 2016. The project was approved by Ethics Committees of Hospital Italiano and IBYME (Protocol #C004-1/2012); patients signed a written informed consent. Ten fresh biopsies (non-tumor and tumor sections, 1 cm^3^ each) from patients diagnosed with infiltrating BC were collected from the surgical piece, placed in RNA Later® and subjected to RNA extraction and subsequent quantitative real-time PCR analysis. In addition, 38 paraffin-embedded tissue samples from patients diagnosed with BC and subjected to transurethral resection or radical cystectomy, were included in the study and analyzed by immunohistochemistry; Supplementary Table 1 summarizes available information on patient gender, age, tumor stage and grade, as well as evidence of metastasis.

### PCR Primers

Primers were designed for endpoint (analytical) and quantitative real time PCR protocols using Primer-BLAST tool (http://www.ncbi.nlm.nih.gov/tools/primer-blast); sequences and expected PCR fragment sizes are listed in Supplementary Table 2. Some primers were previously reported (14, 15).

## Methods

### Bioinformatics

To perform text mining on BC gene-disease associations, the DisGeNET discovery platform (http://www.disgenet.org/) was used. DisGeNET integrates information on human diseases and their genes from expert-curated databases and scientific literature found by means of text-mining approaches (16). Gene-disease associations are ranked according to DisGeNET score, and annotated with DisGeNET gene-disease association type ontology. A set of search terms related to BC was selected to browse all DisGeNET databases and to search for *CDH1* among the output list. The DisGeNET version containing 628,685 associations between 17,549 genes and 24,166 diseases was used in the present study (March, 2019).

Listed somatic mutations in the *CDH1* (E-cadherin) and *CTNNB1* (β-catenin) genes in BC were retrieved from the COSMIC (http://cancer.sanger.ac.uk/cosmic) catalog of public domain data tool. COSMIC provides information about mutations affecting tumor-associated genes, and establishes a hierarchy of the 20 most commonly mutated genes in a specific tumor, as well as number of cases in which a mutation was reported. For each mutation, information is available on position and type of change, as well as amino acid(s) involved; in case of substitutions, it provides information on the specific change that occurs with the mutation. The COSMIC v88 used in this study includes over 6 million coding mutations across than >1.4 million tumor samples curated from >26,000 publications (17). Information was filtered to obtain data from samples of patients diagnosed with transitional cell carcinoma of the bladder, which were 5,774 of the total 8,359 BC samples (March, 2019).

### MB49 and MB49-I Cell Culture and Orthotopic Tumors

MB49 and MB49-I cell lines were cultured as previously reported (11). At 70–80% confluence, cells were harvested and processed for RNA and protein (Western immunoblotting/immunocytochemistry) analyses.

MB49 and MB49-I orthotopic tumors were generated as previously described (11). Basically, orthotopic cell injection in C57BL/6 female mice was performed by placing a catheter into the urethra. Previously, focal electrocautery was performed to induce punctual damage to the bladder wall. MB49 and MB49-I suspensions from subconfluent cell culture monolayers were instilled in 100 μl RPMI 1640 medium. Mice were monitored twice weekly for hematuria and by bladder palpation. Two weeks after tumor cell inoculation, the animals were sacrificed and tumors included in paraffin for subsequent immunohistochemical analysis.

### Transient Transfection Assays

MB49-I cells transient transfection was performed using Lipofectamine® 2000 (Thermo) and 100 pmol/ml of a specific small interfering Ephrin-B1 RNA (siRNA) (#39437, SCB; pool of 3 target-specific 19–25 nt siRNAs designed to knock down gene expression) following a standard procedure (18). Control assays with scramble siRNA (#37007, SCB) were included.

### RNA Extraction, cDNA Synthesis, Standard, and Quantitative Real Time PCR

Cell and tissue total RNA was isolated and processed for complementary DNA (cDNA) synthesis (14, 15, 18). PCR samples were prepared with SYBR Green® PCR Master Mix (Thermo) mixture and run in CFX96 Touch™ (BioRad) PCR unit. Triplicate samples and negative controls (no template) were always included. Dissociation curves were run to confirm signal specificity. GAPDH was selected as endogenous control and MB49 cells as reference. The calculation describing these relations is 2^−ΔΔCt^, where ΔΔCt = [ΔCt test sample – ΔCt reference sample], and ΔCt = [Ct gene under study – Ct endogenous gene]. Alternatively, calculations were done using 2^−ΔCt^ method, where ΔCt = [Ct gene under study – Ct endogenous gene]. Primer sequences and expected PCR fragment sizes are listed in Supplementary Table 2.

### Protein Extraction and Western Immunoblotting Protocols

Protein extraction and analysis was done essentially as previously described (14, 15, 18). Basically, cell monolayers were incubated with PBS on ice, followed by protein extraction with RIPA (20 mM Tris-HCl, pH = 7.5, 150 mM NaCl, 1% NP-40, 1% sodium deoxycholate, and standard protease inhibitors cocktail) buffer. For Stat3 and phospho-Stat3 detection, buffer was supplemented with 1 mM orthovanadate, 10 mM β-glycerol phosphate, and 25 mM sodium fluoride (phosphatase inhibitors). Cells were harvested using a scrapper, and lysis was completed by physical rupture with a 22G needle syringe. Cell lysates were centrifuged at 13,000 × *g* for 30 min, pellets were discarded, and protein content was quantified using the Bradford method with a commercial reagent (BioRad) following manufacturer's instructions.

The NE-PER Nuclear and Cytoplasmic Extraction Reagents (Pierce Protein Research Products (Thermo) commercial kit was used to obtain MB49 and MB49-I cell cytoplasmic and nuclear extracts.

Proteins were separated by electrophoresis in 10% Sodium Dodecyl Sulfate-PolyAcrylamide Gel Electrophoresis (SDS-PAGE) followed by Western immunoblotting. Proteins were immunodetected using standardized protocols and specific antibodies. Protein bands obtained in development plates (AGFA, Belgium) were digitized using a scanner (Hewlett-Packard, USA) and, in some cases, the signal density was quantified with the Image J software (Wright Cell Imaging Facility; Toronto, ON, Canada), and normalized with loading controls.

### Fluorescence Immunocytochemical Analysis

Cells grown on glass coverslips were fixed and subjected to immunocytochemical analysis (14, 15, 18). Cell nuclei were stained with 1 μg/ml Hoechst 33342 or 5 μg/ml propidium iodide (BD Pharmigen) in PBS for 5 min, as specifically indicated. Negative controls were done placing purified mouse or rabbit IgG at the same concentration of primary antibody. Presence of filamentous actin (F-actin) was detected with AlexaFluor 488 phalloidin (Thermo). Stained cells were observed in a fluorescence microscope (Nikon-C2, excitation filters 488 nm/544 nm, emission filters 523–530 nm/570-lp nm); when required, images were analyzed using the Image J software.

### Immunohistochemical Analysis

Immunohistochemical analysis was performed in paraffin-embedded sections from orthotopic tumors of MB49 and MB49-I cell lines, normal C57BL/6 mice bladder and human bladder tumors. Five μm-tissue sections were stained with hematoxylin/eosin for cell morphology analysis or subjected to immunohistochemistry of E-cadherin, β-catenin, pSer33-β-catenin, and Ephrin-B1. In negative controls, primary antibody was replaced by IgG added at the same concentration of primary antibodies. Signal was developed with LSAB+System-HRP (Dako) colorimetric system. Ephrin-B1 staining was scored by two anatomo-pathologists, according to the percentage of stained cells (0, no stained cells, 1, 1 to 29%, 2, 30 to 69%, 3, 70 100% stained cells) and the intensity of the signal (0, without staining; 1: weak; 2: moderate; 3: strong), and a total score was calculated (≥3, “high” Ephrin-B1; <3 “low” Ephrin-B1). E-cadherin staining score was normal (≥1–29% cells with a membrane signal) or abnormal (low or high signal, but without membrane localization).

### Cell Migration and Invasion Assays

Cell migration and invasion assays were run in MB49-I cells transiently transfected with a siRNA toward Ephrin-B1 and Control (scramble SiRNA). The procedure was done basically as previously reported (18) (8 h to assess migration and 40 h to evaluate invasion). In the lower part of the device, medium with 10% FBS was placed to act as chemoattractant. Cells were fixed and stained with 0.5% violet crystal solution in 20% methanol. Images from six different fields were recorded and cells counted using the Image J software.

### Statistical Analysis

All experiments were run in triplicates. Results are expressed as mean ± SEM. Statistical analysis was done using GraphPad Prism software, version 5.01 (GraphPad Software, USA) and InfoStat, version 2015 (InfoStat Group, Argentina). For assays involving two groups, the Mann Whitney or Wilcoxon test was performed, as required; for assays involving >2 groups, the Kruskal Wallis test followed by multiple decompositions according to Dunn was run. Analysis of Ephrin-B1 and E-cadherin immunohistochemistry results was done using the Pearson Chi-Square test. A *P* < 0.05 was considered statistically significant.

## Results and Discussion

### Bioinformatics Survey of BC and E-Cadherin

**Figure 1:**
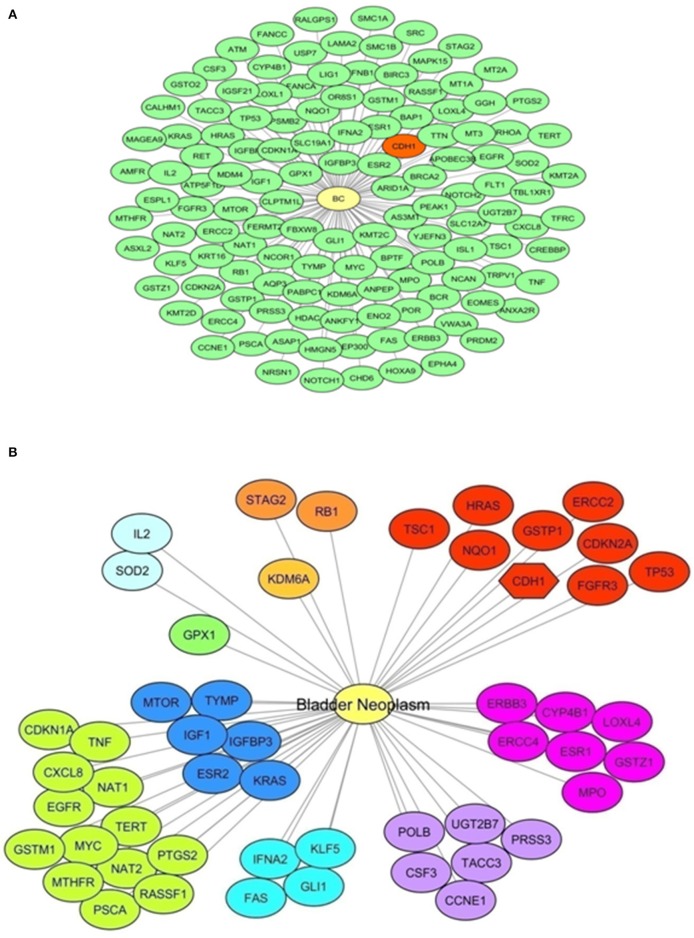
E-cadherin and Bladder Cancer. A bioinformatics survey. **(A)** Network representation of genes annotated to the terms “Malignant neoplasm of urinary bladder,” “Bladder Neoplasm,” “Transitional cell carcinoma of bladder,” “Carcinoma *in situ* of bladder,” and “Carcinoma of bladder” obtained with DisGeNET using Cytoscape. A yellow node represents the “Bladder Cancer” disease node. Green nodes represent disease-related genes (130 genes). In red, the *CDH1* gene is highlighted. **(B)** Network representation of genes annotated to the term “Bladder Neoplasm” obtained with DisGeNET using Cytoscape. A yellow node represents the Bladder Cancer disease node. Other nodes represent disease-related genes, showing same colors for equal score. Red colored nodes depict the highest score (0.6); the *CDH1* gene is among these genes and highlighted with a different node shape. Information on the score and association type is presented in Supplementary Table 3.

A bioinformatics analysis was done to retrieve a list of BC-associated genes. To perform this analysis, the DisGeNET database was surveyed to identify genes annotated to the terms “Malignant neoplasm of urinary bladder,” “Bladder Neoplasm,” “Transitional cell carcinoma of bladder,” “Carcinoma *in situ* of bladder,” and “Carcinoma of bladder.” One hundred and thirty genes were identified; *CDH1* was among genes with highest score (*CDH1, CDKN2A, ERCC2, FGFR3, GSTP1, HRAS, NQO1, TP53, TSC1*), specifically when only the term “Bladder neoplasm” was surveyed. Figure 1 shows a graphical representation of both networks, and Supplementary Table 3 lists genes retrieved from DisGeNet survey, with their association type and score.

In addition, a search of *CDH1* somatic mutations in BC was done using the COSMIC bioinformatics tool. As a result, *CDH1* mutations were found in 17/587 (2.90%) tested BC samples, 15 of which were located in exonic sequences of different protein segments [residues 9, 27, 28, 150, 159, 210, 240, 248, 357, 638 (twice), 641, 677, 731, 785]. Table 1 lists the identified mutations.

**Table 1:** *CDH1* somatic mutations and BC.

**Transcript**	**Sample ID**	**AA mutation**	**CDS mutation**
ENST00000261769	2727096	p.S9*	c.26C > A
ENST00000261769	2726284	p.P27fs*27	c.79_85delCCCTGCC
ENST00000261769	2725343	p.C28fs*1	c.84_85delCC
ENST00000261769	2721157	p.R150K	c.449G > A
ENST00000261769	2720314	p.P159 > HT	c.475_476insATA
ENST00000261769	2714567	p.E210Q	c.628G > C
ENST00000261769	2721980	p.I248N	c.743T > A
ENST00000261769	2719872	p.?	c.1320 + 2T > G
ENST00000261769	2772700	p.?	c.1320 + 5G > A
ENST00000261769	2719775	p.K557N	c.1671G > C
ENST00000261769	2724773	p.W638*	c.1914G > A
ENST00000261769	2097275	p.W638*	c.1914G > A
ENST00000261769	2722509	p.Q677*	c.2029C > T
ENST00000261769	2097330	p.L731F	c.2191C > T
ENST00000261769	992969	p.N785N	c.2355C > T
ENST00000261769	1995691	p.N240Xfs*10	c.?
ENST00000261769	2395174	p.Q641*	c.?

*A bioinformatics analysis using COSMIC. Data retrieved from the COSMIC database (GRCh38 · COSMIC v90)*.

Altogether, these studies showed evidence of *CDH1*/E-cadherin involvement in BC, which was related only in few cases to mutations in the *CDH1* gene. Results of these surveys encouraged authors to further investigate changes in E-cadherin and related molecules, and the underlying mechanisms in BC.

### Expression of E-Cadherin and EMT-Related Markers in MB49 and MB49-I Murine BC Cell Lines

**Figure 2:**
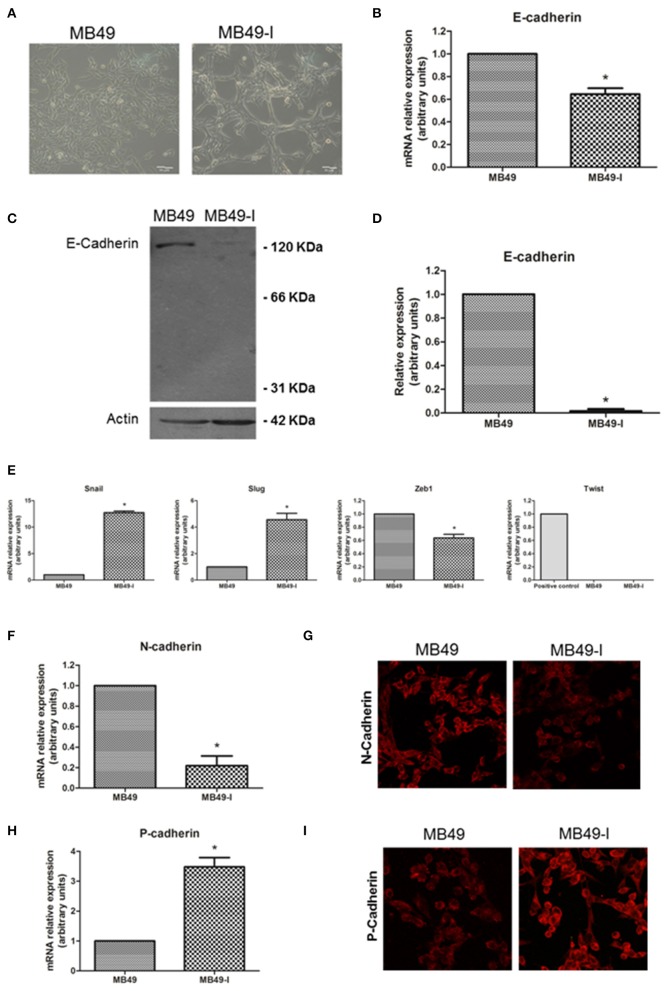
EMT molecules and BC. Studies in the MB49 and MB49-I cell murine models. **(A)** Phase contrast microscopy of MB49 and MB49-I cells. Graph Bar = 20 μm. **(B)** Quantitative analysis of E-cadherin mRNA in MB49 and MB49-I cells by real-time PCR. Results are plotted as mean relative expression with Standard Error of the Mean (SEM); *P < 0.05. **(C)** Immunodetection of E-cadherin protein forms in total protein extracts of MB49 and MB49-I cells after performing 10% polyacrylamide gel electrophoresis (SDS-PAGE) followed by Western immunoblotting with anti E-cadherin antibody (610181; 2.5 μg/ml). Actin (I-19; 2 μg/ml) was used as loading control. References indicate the molecular weight standards of 66 and 31 KDa and the 120 KDa E-cadherin full length form. **(D)** Densitometric analysis of results obtained by Western Immunoblotting for the expression of E-cadherin performed with the ImageJ program; results are shown as percentage of the expression level of CadE in line MB49-I. Assessments of 3 replicates were included in the analysis. Data is shown as mean ± SEM. **(E)** Quantitative analysis by real-time PCR of Snail, Slug, Zeb1, and Twist mRNA expression in MB49 and MB49-I cells. For Twist, mouse testis was used as a positive control. ns, not significant; *P < 0.05. **(F)** Quantitative analysis by real-time PCR of N-cadherin mRNA expression in MB49 and MB49-I cells. **(G)** Fluorescence immunocytochemistry analysis by confocal laser microscopy of N-cadherin (H-63; 2 μg/ml) in MB49 and MB49-I cells. Negative controls are shown on the right, and cell nucleae were visualized using HOESCHT 33342. **(H)** Quantitative analysis by real-time PCR of P-cadherin mRNA expression in MB49 and MB49-I cells, *P < 0.05. In all cases, relative expression levels of each mRNA was calculated using actin as endogenous gene and the MB49 cell line as reference. **(I)** Fluorescence immunocytochemistry analysis by confocal laser microscopy of P-cadherin (H-105; 2 μg/ml) in MB49 and MB49-I cells. Negative controls are shown on the right, and cell nucleae were visualized using HOESCHT 33342.

Initial studies were focused on evaluating the expression of E-cadherin and some EMT-related genes in MB49 and MB49-I *in vitro* cell cultures (Figure 2A). While in standard RT-PCR assays E-cadherin mRNA was detected in both cell lines, quantitative real-time PCR analyses revealed a 30% decrease (*P* < 0.05) in E-cadherin transcript levels in MB49-I cells compared to MB49 parental cells (Figure 2B). In agreement with these findings, E-cadherin protein analysis by Western immunoblotting revealed significant lower expression levels of the 120 KDa full-length adhesion full-length protein form in MB49-I cells, representing <10% of those found in MB49 cells (Figures 2C,D). These findings are consistent with early studies done in human samples, in which lower E-cadherin levels were found in infiltrating than in superficial bladder tumors (2, 19, 20). In any case, while a 30% decrease in the *CDH1* transcript was found in MB49-I compared to MB49 cells, a sharp decrease to undetectable protein levels was determined. Differences observed between both cell lines in E-cadherin transcript and protein levels may relate to expression levels of the adhesion protein present in these cell lines and methodologies used to detect them. Both cell lines depict low E-cadherin mRNA expression levels (around Ct 36), but the quantitative real time PCR procedure can accurately measure low transcript levels in both cell lines. On the other hand, expression levels of the adhesion protein may be close to the limit of detection, resulting in a very poor/absent signal in MB49-I cells, even when using sensitive detection systems. Alternatively, differences between RNA and protein levels in the murine cell lines may result from selective regulatory post-transcriptional, translational and post-translational mechanisms, i.e., pre-mRNA splicing, polyadenylation, capping, and mRNA transport, turnover, storage, and translation (21). Some post-transcriptional mechanisms have been shown to critically influence EMT (22–24). Among mechanisms determining mRNA turnover by ribonucleoproteins, HuR is one of the best-studied regulators of cytoplasmic mRNA fate (21), and evidence supports its interaction with the 3'-untranslated region of E-cadherin mRNA and regulation of E-cadherin translation (25). In BC, HuR appears to regulate the expression of many urinary tumors-associated molecules (26), and a significant association between positive cytoplasmic HuR signal and high tumor grade, pT stage and micro-vessel density has been reported in BC (27). Differences found between E-cadherin expression levels in both cell lines and between mRNA and protein levels found may be, at least in part, related to the expression of HuR in the murine cell model and modulation of E-cadherin mRNA and protein stability. Mouse cells have been reported to conserve consensus sequences and mechanisms found in humans for these regulations (28). In addition to this regulatory mechanism, the translated E-cadherin protein may be subjected to proteolytic cleavage, catalyzed by matrix metalloproteinases (MMP-3, MMP-7, MMP-9, and MT1-MMP), A-disintegrin-and-metalloproteinases (ADAM10 and ADAM15), plasmin, and kallikrein 7, releasing the 86 KDa ectodomain fragment and a 38 KDa fragment associated to the membrane, which is degraded to smaller fragments (5, 29). Since MMP9 has been previously reported in BC (30) and in the MB49-I cell model (11), part of the lower signal may be the result of E-cadherin processing; E-cadherin cellular fragments may not be resolved in 10% SDS-PAGE gels, are below the limit of the detection, or lack the antibody epitope, and are not shown in the profile.

Since transcriptional repression is one mechanism associated to decreased E-cadherin (5), expression of Snail, Slug, Zeb1, and Twist transcriptional repressors was analyzed in both cell lines. As shown in Figure 2E, an increased expression (*P* < 0.05) of Snail and Slug transcripts was observed in MB49-I cells compared to MB49 cells. On the other hand, while lower levels (*P* < 0.05) of Zeb1 mRNA were detected in MB49-I cells compared to those in MB49 cells, no detectable levels of Twist were found in either BC cell line. Findings in the present report are in line with a previous study by Gou et al. (31), describing a relationship between a higher Snail expression, and the presence of advanced tumors among 332 human samples from patients with superficial BC evaluated by immunohistochemistry; in the same report, Snail was a predictor of disease recurrence and progression. Moreover, a higher expression of Slug was also previously reported in infiltrating tumors, in a study of 47 patients diagnosed with BC (32). Finally, Tang et al. (33) reported significantly lower expression levels of Twist1 mRNA in 13 paired urothelial bladder cancer specimens than the non-tumor mucosas.

The cadherin switching phenomenon represents an important aspect of the EMT, and it has been typically described as a process by which E-cadherin expression is replaced by N-cadherin expression (34, 35). However, later studies found an abnormal expression of other members of the superfamily as part of the cadherin switch (36). Thus, the E-cadherin to N-cadherin and/or P-cadherin switch phenomenon was analyzed in the *in vitro* murine BC model. Regarding N-cadherin, mRNA expression analysis revealed significant lower levels of this transcript in MB49-I compared to MB49 cells (Figure 2F). In line with these findings, fluorescence immunocytochemistry revealed a lower signal for N-cadherin in MB49-I compared to MB49 cells (Figure 2G). Previous studies have reported ~40% of bladder tumors depicting a positive signal for this protein (37–39). This percentage was found in either NMIBC (37) or MIBC (40) tumor cohorts, showing that not all invasive tumors show increased expression of N-cadherin. While some of these studies showed an association between increased N-cadherin expression and recurrence-free survival (37, 38), others failed to find a relationship between N-cadherin expression with clinicopathological parameters (39, 41). In line with the results of the present report, a study by Jäger et al. (42) found a positive N-cadherin expression in superficial (Ta/T1) cases to be a risk factor, and a lack of expression in MIBC cases correlated with reduced patient survival. Moreover, they reported 14% (13/92) N-cadherin-negative (2 × T2, 8 × T3, and 3 × T4) MIBC, and 12/13 patients died of BC within 18 months; the average survival time in this group was 3.5-fold lower than in MIBC cases expressing N-cadherin. In another study, the N-cadherin-negative muscle-invasive tissue samples (“high risk”) depicted a 2-fold higher expression level of the VEGF angiogenic factor than N-cadherin-positive muscle invasive cancers (43), highlighting the association between negative N-cadherin expression in BC and tumor aggressiveness.

With regard to P-cadherin assessment, transcript (Figure 2H) and protein (Figure 2I) levels were higher (*P* < 0.05) in MB49-I cells compared to MB49 cells. Results from this report agree with several studies that show P-cadherin expression in association with BC progression. Rieger-Christ et al. (44) described P-cadherin in over 90% of the cases in a cohort of ~100 tumors; authors also reported N-cadherin expression, absent in normal urothelium but localized to the membrane in focal areas of the tumor mass in invasive tumors. Later, Bryan et al. (45) studied over 150 bladder tumors, finding increased membranous P-cadherin expression in almost half of all MIBC, accompanied by significantly lower expression of E-cadherin. Increased P-cadherin expression was associated with worse BC-specific survival, and P-cadherin status was an independent prognostic factor (alongside grade and stage). Moreover, functional *in vitro* experiments showed that altering the balance of E- and P-cadherin in favor of P-cadherin expression enhanced anchorage-independent growth, and P-cadherin alone was unable to mediate normal cell–cell adhesion. Authors concluded that P-cadherin expression promoted a more malignant and invasive phenotype of BC, even in the presence of E-cadherin. The same year Mandeville et al. (46) reported the expression of P-cadherin in BC, and found that P-cadherin overexpression induced an increase in migratory capacity in *in vitro* models. Van Marck et al. (47) later showed that stable knockdown of P-cadherin in RT-112 BC cells diminished invasion and migration, and promoted intercellular adhesion, supporting a P-cadherin transformation promoting role in BC. Mandeville et al. (46) suggested P-cadherin to have a role in regulating migration of basal cells to the intermediate cell layer in normal urothelium as well as a role in neoplastic progression, while Bryan et al. (45) suggested that P-cadherin may be induced in BC to reduce cell-cell adhesion and to enable cell-matrix interactions as a first step to invasion in a similar fashion to N-cadherin. In Jäger's report (42), high P-cadherin gene expression proved to be an independent favorable prognostic factor of cancer-related survival, and a risk factor of recurrence in superficial cancers after tumor resection. In 2014, Wang et al. (48) reported a correlation between P-cadherin high expression and tumor progression. Finally, using a molecular approach, a thorough report by Choi and collaborators described the identification of distinct basal and luminal subtypes of MIBC with different sensitivities to frontline; in the study, *CDH3*, the gene encoding P-cadherin was listed among gene classifiers for muscle invasive progression and muscle invasive overall survival (49).

### Protein Assessment of the Adherent Complex Members. β-Catenin Expression Analysis

**Figure 3:**
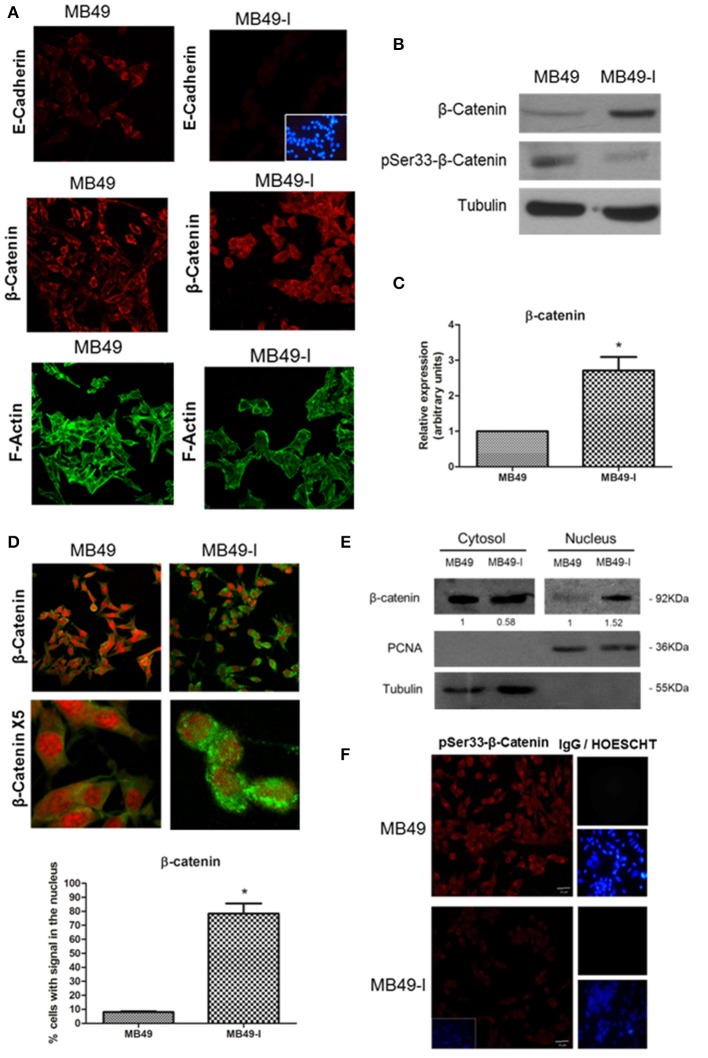
Evaluation of adherent complex members in MB49 and MB49-I cells. **(A)** Fluorescence immunocytochemistry of E-cadherin (610181; 2.5 μg/ml), (top panels) and β-catenin (610153; 2.5 μg/ml) (middle panels) and analysis by confocal microscopy. Bar indicates 20 μm (Magnification 40×). Negative controls are shown on the right; cell nucleae were visualized using HOESCHT 33342. Confocal laser microscopy analysis of actin cytoskeleton organization by visualization of filamentous actin (F-actin) labeled with phalloidin-Alexa488 in MB49 and MB49-I cells (bottom panels). **(B)** Immunodetection of β-catenin (610153; 2.5 μg/ml) and pSer33-β-catenin (Ser33-R; 4 μg/ml) after performing 10% SDS-PAGE followed by Western immunoblotting of MB49 and MB49-I cell extracts. Loading controls were done using a specific antibody toward β-tubulin (D-66; 0.05 μg/ml). **(C)** Densitometric quantification analysis of β-catenin in MB49 and MB49-I cells using the Image J software; *P < 0.05. **(D)** Fluorescence immunocytochemistry of β-catenin (610153; 2.5 μg/ml) and analysis by confocal microscopy in MB49 and MB49-I cells. Cell nuclei were visualized using Propidium Iodide. Bar = 20 μm (Magnification 40×). MB49-I cells with nuclear localization of β-catenin are indicated with an arrow. Graphic representation of the percentage of MB49 and MB49-I cells with β-catenin signal in the nucleus; *P < 0.05. **(E)** Detection of β-catenin (610153; 2.5 μg/ml) after 10% SDS-PAGE followed by Western immunoblotting of cytoplasmic and nuclear fractions of MB49 and MB49-I cell extracts. Cell fraction loading and purity: β-tubulin (positive in the cytoplasmic fraction; D-66, 0.05 μg/ml), PCNA (positive in the nuclear fraction; PC10, 0.2 μg/ml). **(F)** Fluorescence immunocytochemistry and confocal laser microscopy analysis in MB49 and MB49-I cells of pSer33-β-catenin (Ser33-R; 4 μg/ml). Negative controls shown on the right; cell nucleus visualized using HOESCHT 33342.

Considering the relevance of the adhesion complex integrity upon cell-cell adhesiveness, and the decreased E-cadherin mRNA and protein levels found in MB49-I cells, subcellular localization of some members of the adherent complex was evaluated. Localization of E-cadherin and β-catenin was determined in both cell lines by fluorescence immunocytochemistry, while evaluation of the actin cytoskeleton organization was performed by Filamentous actin (F-actin) staining with Alexa488-phalloidin. As shown in Figure 3A (top panels), immunolocalization analysis of E-cadherin in whole cells revealed a signal in the cytoplasm of both cell lines, but ~50% less intense in MB49-I cells, in line with the results of mRNA and total E-cadherin protein expression analysis described above (Figure 2C). On the other hand, β-catenin immunolocalization analysis revealed striking differences between both cell lines; while in MB49 cells the β-catenin signal was localized mainly in the cytoplasm, in MB49-I cells it was stronger and distributed in the cytoplasm and nucleus (Figure 3A, middle panel). F-actin fluorescence distribution evidenced stress fibers in both lines (Figure 3A, bottom panels).

To confirm a higher expression of β-catenin protein in MB49-I cells, Western immunoblotting of total protein extracts from both cell lines was done. In agreement with the immunocytochemistry results, higher levels of β-catenin were detected in MB49-I cells compared to MB49 cells (Figures 3B,C). These changes could not be attributed to an increase in mRNA expression (MB49: 1; MB49-I: 0.978±0.043; ns). Based on these findings, β-catenin subcellular distribution was quantified, finding nuclear localization in ~80% of MB49-I cells and in ~10% of MB49 cells (Figure 3D). This evaluation was also done in cytoplasmic and nuclear protein extracts (β-tubulin cytoplasmic marker; PCNA nuclear marker). As shown in Figure 3E, β-catenin signal in MB49-I cells was weaker in the cytoplasmic fraction and stronger in the nuclear fraction, when compared to MB49 cells.

In normal cells, β-catenin localizes at the cell membrane in a complex with E-cadherin; cytoplasmic β-catenin is maintained at low levels, being constitutively captured by a destruction complex that facilitates its N-term phospho targeting, ubiquitination and destruction in the proteasome. The adenomatous polyposis coli (APC) and Axin are structural components of the destruction complex, while CK1 casein kinases and 3 glycogen synthase kinase-3β (GSK-3β) are recruited to serine phosphorylate β-catenin. Under certain conditions, β-catenin accumulates in the cytosol and is shuttled to the nucleus, where it binds and forms complexes with transcription factors of the lymphoid enhancer factor (Lef)/T-cell factor (TCF) family, and activates target genes, which are regulators of cell proliferation and involved in tumorigenesis. N-terminal β-catenin phosphorylation is required for its degradation and for attenuating its effect on transcription (50–52). β-catenin nuclear localization has been related to tumor progression and aggressiveness in several tissues (i.e., breast, renal, and colorectal cancer); in particular, there are reports describing an aberrant localization of β-catenin in the cell cytoplasm and nucleus in BC (53, 54).

Taking into account that β-catenin is phosphorylated in the degradation complex by GSK-3β in Ser33, Ser37, and Ser41 (52, 55), presence and localization of pSer33-β-catenin were assessed in MB49 cells and MB49-I cells, both by Western immunoblotting and fluorescent immunocytochemistry using a commercial antibody. As result, lower levels of P-Ser33-β-catenin were found in MB49-I cells compared to MB49 cells (Figures 3B,F, respectively), in agreement with β-catenin localization found in the MB49-I cell nucleus. In line with these findings, Roy et al. (56) reported the results of next generation sequence analysis done in 15 primary bladder adenocarcinomas, in which mutations were found in *CTNNB1* (encoding β-catenin) (3/15, 20%), including activating missense mutations at known hotspots (p.D32N and p.S45F) and amplification; in the study, the immunohistochemistry analysis revealed β-catenin nuclear localization in mutated samples. In addition to these findings, the COSMIC database lists 10/29 mutations (all missense) around Ser33, Ser41, and adjacent residues, from 764 samples of transitional cell carcinoma of the bladder evaluated. The list is shown in Supplementary Table 4.

### Expression Analysis of Ephrin-B1 and Related Proteins in MB49 and MB49-I Cells

Studies have identified Ephrin-B1 as a target gene of β-catenin nuclear transcriptional activity (57–59). Moreover, Ephrin-B1 expression has been associated to tumor progression and invasion in cell models (60, 61) and in different cancer types [hepatocarcinoma: (62), ovarian: (63), gastric: (64)]. Other members of the Ephrin family have been studied in BC. Among them, a higher expression of Ephrin-A1 and EphA2 receptor was found in BC cell lines and in advancing tumor compared to controls, as well as an association between their expression and tumor stage (65). Moreover, a high expression of arterial Ephrin-B2 and venous EphB4 receptor was reported in BC, and proposed to contribute to tumor angiogenesis (66).

**Figure 4:**
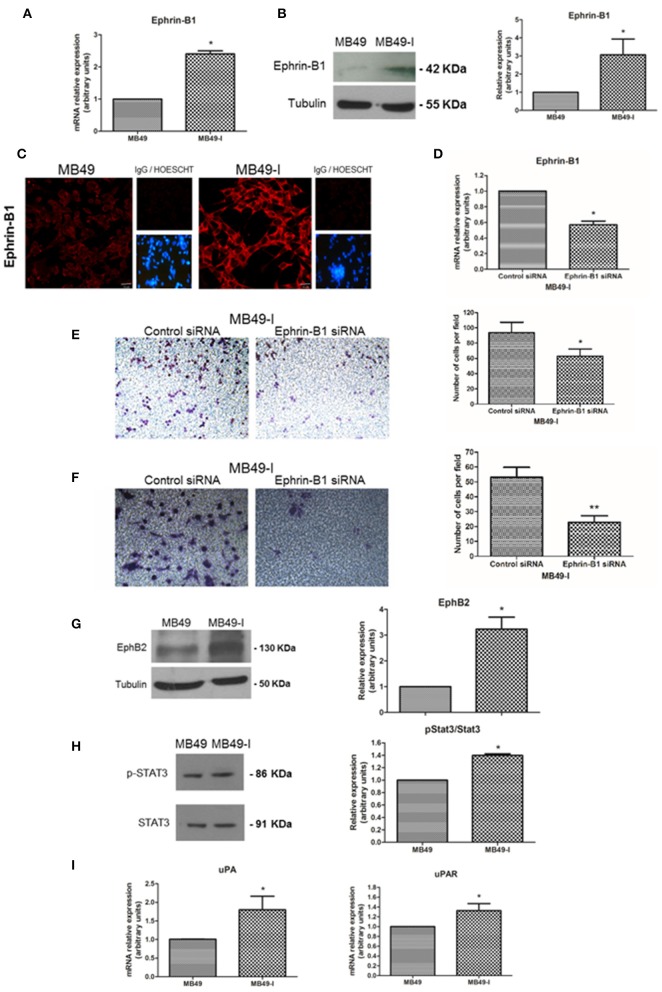
Expression analysis of Ephrin-B1 and related proteins in the MB49 and MB49-I cell murine models. **(A)** Quantitative expression analysis of Ephrin-B1 mRNA in MB49 and MB49-I cells by real-time PCR. (**B**, Left) Immunodetection of Ephrin-B1 (A-20; 2 μg/ml) after 10% SDS-PAGE of MB49 and MB49-I cells protein extracts followed by Western Immunoblotting. β-tubulin (D-66, 0.05 μg/ml) used as loading control. (Right) Densitometric analysis of Ephrin-B1 immunodetection; *P < 0.05. **(C)** Fluorescence immunocytochemistry and analysis by confocal laser microscopy of Ephrin-B1 (A-20; 2 μg/ml) in MB49 and MB49-I cells. (Right) Negative controls; cell nucleae were visualized using HOESCHT 33342. **(D)** Quantitative analysis of Ephrin-B1 mRNA by real-time PCR in cells transiently transfected with an Ephrin-B1specific siRNA (cells MB49-I siRNA Ephrin-B1) or control siRNA (MB49-I siRNA Control cells); *P < 0.05. (**E**, Left) Representative images of the migration assay obtained by phase contrast microscopy (Magnification 20×). (Right) Graphical representation of the migration assay results in siRNA MB49-I Ephrin-B1 and siRNA control MB49-I cells. The average number of cells in each of the evaluated fields is plotted; *P < 0.05. **(F**, Left). Representative images of the invasion assay obtained by phase contrast microscopy (Magnification 20×). (Right) Graphic representation of the invasion assay results. The average number of cells in each of the evaluated fields is plotted; **P < 0.001. (**G**, Left) Immunodetection of EphB2 (H-80; 2 μg/ml) in MB49 and MB49-I cells protein extracts after 10% SDS-PAGE/Western Immunoblotting. Immunodetection of β-tubulin was carried out as loading control. (Right) Densitometric analysis of EphB2; *P < 0.05. (**H**, Left) Immunodetection of pStat3 (C-20; 2 μg/ml) and Stat3 (B-7; 2 μg/ml) of protein extracts of MB49 and MB49-I cells, after 10% SDS-PAGE/Western Immunoblotting. Loading control: β-tubulin (D-66, 0.05 μg/ml). (Right) Densitometric analysis of phospho-Stat3 immunodetection. Signal intensity of this molecule was normalized using Stat3 signal intensity and MB49 cells as reference; *P < 0.05. **(I)** Quantitative analysis of uPA and uPAR mRNA by real-time PCR in MB49 and MB49-I cells; *P < 0.05.

Based on this background information, Ephrin-B1 mRNA and protein levels were determined in both cell lines, finding significantly higher expression of Ephrin-B1 transcript in MB49-I cells than in MB49 cells (Figure 4A). In agreement with these results, a stronger signal for Ephrin-B1 was found in MB49-I than in MB49 protein cell extracts (Figure 4B). This increase was also evidenced by fluorescence immunocytochemistry, with an Ephrin-B1 signal >300% higher in MB49-I cells than in MB49 cells (Figure 4C). To evaluate the association between Ephrin-B1 expression and an invasive cellular behavior, MB49-I cells were transfected with Ephrin-B1 (MB49I-siRNAEphrin-B1 cells) or scramble (MB49I-siRNAControl) siRNA. The effectiveness of the procedure was confirmed by a decrease in Ephrin-B1 mRNA levels (Figure 4D). MB49-I siRNA Ephrin-B1 treated cells depicted lower migratory and invasive capacity than control cells (Figures 4E,F).

Co-expression of Ephrin-B1 and the EphB2 receptor has been observed in small cell lung cancer (67), gastric cancer (64) as well as in medulloblastoma (61). Also, their co-expression was related to metastasis by immunohistochemistry in a study done in 50 cholangiocarcinoma samples (68). Based on these findings, EphB2 expression was evaluated in total protein cell extracts, finding 3 times higher levels in MB49-I cells than in MB49 cells (Figure 4G). These findings are in agreement with the association reported between high EphB2 expression and EMT induction and progression of human cervical cancer (69), although they contrast with a report describing its decrease in human BC samples (70).

Among several signal transduction mechanisms, Ephrin-B1 interaction with EphB2 receptor results in its activation, which is followed by Stat3 recruitment, phosphorylation and enhanced transcriptional activation (71). Deregulation of Stat3 signaling has been reported in several solid tumors, among them BC (72), and its activation has been associated to tumor invasion and metastasis (73). Thus, the expression of Stat3 and the Tyr705-phospho Stat3 (pStat3) activated form was analyzed in both cell lines, finding a 35% increase in pStat3/Stat3 ratio in MB49-I cells (Figure 4H). Moreover, the expression of Stat3 target genes, specifically Urokinase-type Plasminogen Activator (uPA) and its receptor uPAR (Urokinase-type Plasminogen Activator Receptor) was evaluated, finding higher levels of both transcripts in MB49-I cells compared to MB49 cells (Figure 4I). In line with these findings, uPA activity was previously reported in MB49-I cells conditioned media (11). These molecules play a fundamental role in tissue remodeling during invasion and metastasis, and their expression was related to BC poor prognosis (74). uPA and uPAR expression has been related to β-catenin transcriptional activity in colorectal tumors (75).

### Expression Analysis of E-cadherin, β-catenin, and Ephrin-B1 in Murine and Human Bladder Tumors

**Figure 5:**
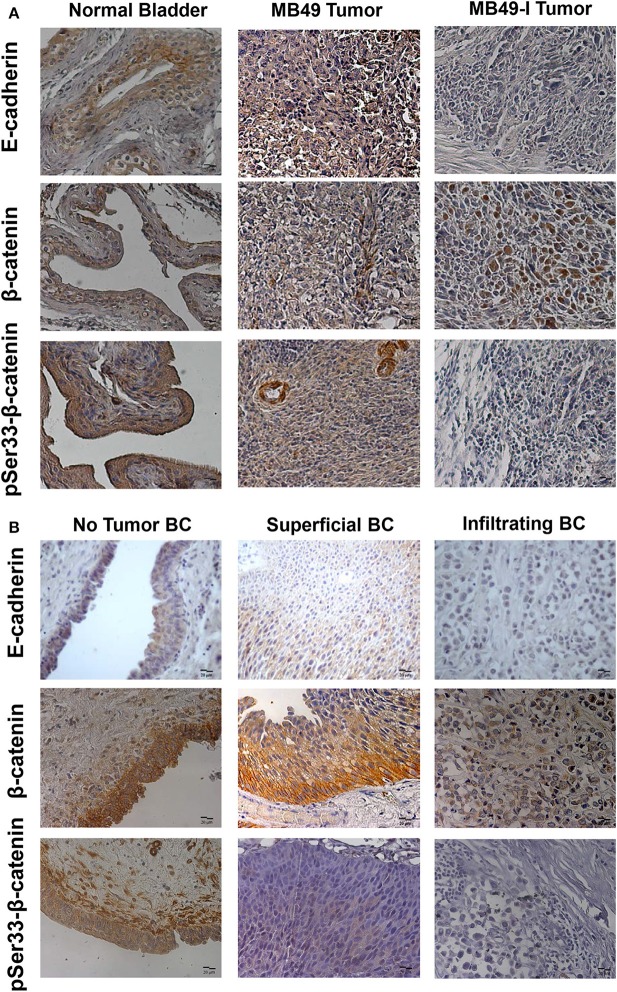
Immunohistochemical analysis of E-cadherin, β-catenin and pSer33-β-catenin in murine and human bladder tissues. **(A)** Colorimetric immunohistochemistry of: Top: E-cadherin (610181; 2 μg/ml), Middle: β-catenin (610153; 2 μg/ml) and pSer33-β-catenin (Ser33-R; 2 μg/ml) in paraffin sections of normal bladder (left), MB49 tumors (middle), and MB49-I tumors (right). Bar = 20 μm (Magnification 40×). Contrast staining was performed using hematoxylin. **(B)** Colorimetric immunohistochemistry of E-cadherin (HECD-1; 0.6 μg/ml), β-catenin (610153; 2 μg/ml), and pSer33-β-catenin (Ser33-R; 2 μg/ml) in paraffin sections of bladder tissue from patients with superficial or infiltrating BC. In addition, a non-tumor section of bladder from patients with infiltrating BC after cystectomy is included. Bar = 20 μm (Magnification 40×). Contrast staining was performed using hematoxylin.

Taking into account findings obtained in cell cultures of the BC murine model, orthotopic tumors were derived from both cell lines, and immunodetection of E-cadherin, β-catenin, pSer33-β-catenin, and Ephrin-B1 was done in paraffin-embedded tissue sections; for all proteins evaluated, murine bladder C57BL/6 mice bladders were included as controls (non-tumor). E-cadherin was immunolocalized at the cell membrane and cytoplasm in normal bladder; contrasting, a cytoplasm signal was mainly found in MB49 tumors, and a low intensity or undetectable signal in MB49-I tumors (Figure 5A, top). β-catenin was immunolocalized at the cell membrane and cytoplasm of normal bladders and MB49 tumors, while MB49-I tumors showed a signal in the nucleus in a high proportion of cells (Figure 5A, middle). Moreover, pSer33-β-catenin immunodetection revealed a strong signal in both normal bladder and MB49 tumors, but very weak in MB49-I tumors (Figure 5A, bottom). In all cases, protein subcellular localization and signal intensity was comparable to that found in both cells growing in monolayers.

A similar analysis done in human tumor samples from patients diagnosed with BC, revealed a strong signal for E-cadherin in the cell membrane of sections of the tumor classified as non-tumor tissue, and a weaker signal in the cytoplasm of tumor sections from superficial and infiltrating tumors, with lowest intensity in the latter (Figure 5B, top), in agreement with previous reports in human BC (2, 19, 20). β-catenin was immunodetected at the cell membrane of non-tumor bladder and superficial tumors, but alterations in its expression and localization were observed as previously reported (19, 20). In particular, a cytoplasmic and nuclear signal was found in a subset of infiltrating tumors evaluated (Figure 5B, middle). This localization of β-catenin was similar to that reported in 87% (26/30) of MIBC evaluated, which was associated it with poor prognostic parameters, among them tumor grade, stage, microvessel density, vascular invasion, and lymph node metastasis (53). For pSer33-β-catenin, a cytoplasmic staining was observed in non-tumoral bladder and superficial tumors, although with less intensity, while no signal was detected in infiltrating tumors (Figure 5B, bottom).

**Figure 6:**
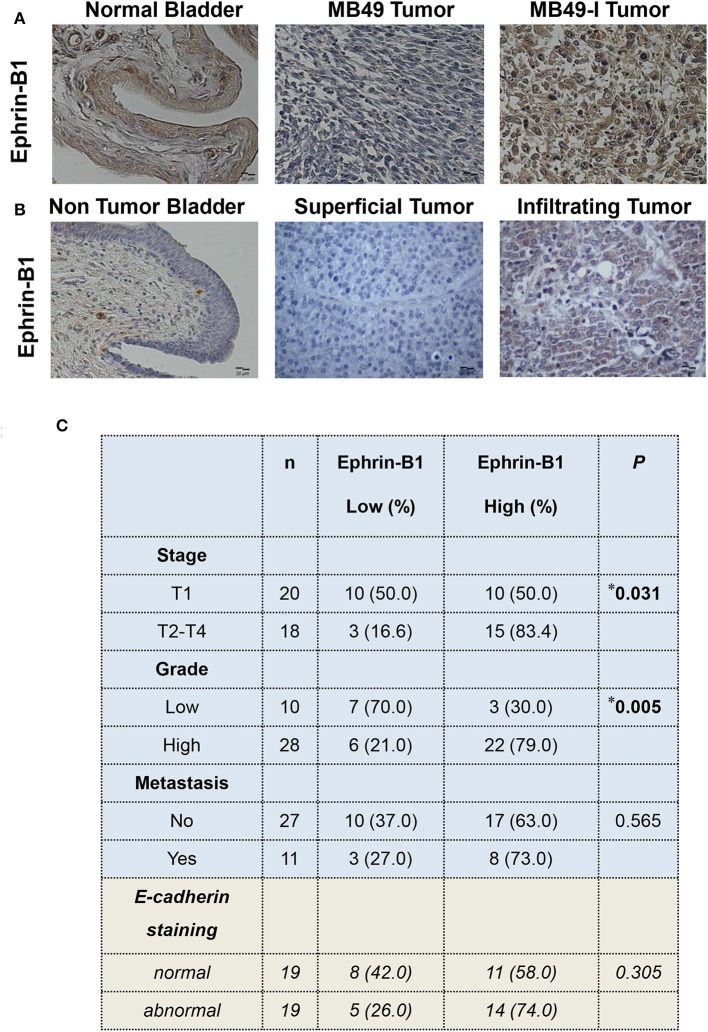
Immunohistochemical analysis of Ephrin-B1 in murine and human bladder tissues. **(A)** Colorimetric immunohistochemistry of Ephrin-B1 (A-20; 2 μg/ml) in paraffin sections of normal bladder (left), MB49 tumors (middle), and MB49-I tumors (right). Bar = 20 μm (Magnification 40×). Contrast staining was performed using hematoxylin. **(B)** Colorimetric immunohistochemistry of Ephrin-B1 (A-20; 2 μg/ml) in paraffin sections of bladder tissue from patients with superficial or infiltrating BC. In addition, a non-tumor section corresponding to bladder of a patient diagnosed with infiltrating BC and undergoing cystectomy is included. Bar = 20 μm (Magnification 40×). Contrast staining was performed using hematoxylin. **(C)** Analysis of the relationship between Ephrin-B1 expression and different clinico-pathological parameters in a set of BC samples. Ephrin-B1 staining analysis was performed assigning a score according to the percentage of stained cells (0, no stained cells, 1:1–29%, 2:30–69%, 3:70–100%) and the intensity of the signal (0, no staining; 1: weak; 2: moderate; 3: strong). The total score was calculated after adding both scores. The *P*-value was calculated using Pearson's Chi square test. *P < 0.05.

Regarding Ephrin-B1 immunodetection, MB49-I tumors depicted a stronger signal compared to normal bladders and MB49 tumors (Figure 6A). To assess Ephrin-B1 expression in human BC, two studies were done. First, fresh biopsies of tumor and non-tumor mucosa were obtained from 10 patients during radical cystectomy. Transcript expression assessed by real-time PCR revealed higher (*P* < 0.05) Ephrin-B1 levels in tumor vs. non-tumor samples (Non tumor = 0.001446 ± 0.0006982, Tumor = 0.003612 ± 0.001740).

Ephrin-B1 immunohistochemical analysis was done in superficial and infiltrating tumors; normal bladder tissue sections found in tumor pieces were also evaluated. A faint or absent signal was observed in the urothelium of the non-tumoral section, while a signal was detected in all tumors (Figure 6B). A low or high Ephrin-B1 score was assigned, according to the percentage of stained cells and the signal intensity. As result, a positive association (*P* = 0.031) was found between Ephrin-B1 expression and tumor stage (Figure 6C), with a high proportion of infiltrating tumors (15/18, 83.4%) depicting a high score. Half of superficial tumors (10/20; 50%) showed a weak signal for Ephrin-B1, but 10 superficial tumors depicted a strong signal, 6 of which were high grade, and other 2 were large tumors with multifocal sites. A positive association was also found between Ephrin-B1 expression and tumor grade (*P* = 0.005), with 79% high-grade tumors depicting a high score. Likewise, 70% low-grade tumors had a low score. No significant relationship was found between Ephrin-B1 staining and the occurrence of metastasis.

E-cadherin expression was also analyzed in the 38 samples, and compared with Ephrin-B1 results, finding 74% tumors depicting an abnormal E-cadherin expression and high Ephrin-B1 expression, although no significant relationship was found between both proteins (Figure 6C). No significant relationship was also found between E-cadherin signal and tumor stage, grade, or metastasis (Supplementary Table 5).

Altogether, findings reported in this investigation further characterized changes in E-cadherin and EMT-related molecules in BC. Using a combination of bioinformatics, a murine model of tumor aggressiveness and BC patient samples, the expression of Ephrin-B1 and related proteins is reported for the first time in association to BC progression/aggressiveness, gaining knowledge on the underlying mechanisms of BC. Evaluation of Ephrin-B1 in a multicenter study with a large cohort of patients will help confirming its role as a biomarker of BC aggressiveness. Since antibodies, recombinant proteins, and peptides developed to target the Ephrin/Eph ligand-receptor system have been reported to reduce tumor growth in breast, prostate, colon, head, and neck cancer animal models and some molecules are under investigation in clinical trials (76), additional studies with these molecules in BC will contribute to determine Ephrin-B1 role as therapeutic target of disease progression.

## Data Availability Statement

The raw data supporting the conclusions of this article will be made available by the authors, without undue reservation, to any qualified researcher.

## Ethics Statement

The studies involving human participants were reviewed and approved by Ethics Committees of Hospital Italiano and IBYME (Protocol #C004-1/2012). The patients/participants provided their written informed consent to participate in this study. The animal study was reviewed and approved by Institute of Oncology Angel H. Roffo Review Board (#2012/02).

## Author Contributions

MV-L, LL and MM were involved in study conception and design and drafted the manuscript. MM, LL, MR, MB, DB, MI, SV, CL, AE, JT, MG, MZ and MV-L participated in data collection. MM, LL, MR, MB, and MV-L participated in data analysis. MV-L was responsible of grants that supported this study, coordinating and supervising the entire project. All authors read and accepted the final version.

### Conflict of Interest

The authors declare that the research was conducted in the absence of any commercial or financial relationships that could be construed as a potential conflict of interest.

## Footnotes

^1^International Agency for Research on Cancer; World Health Organization. http://globocan.iarc.fr.
