# Supplementary material for: Ephrin-B1 Is a Novel Biomarker of Bladder Cancer Aggressiveness. Studies in Murine Models and in Human Samples
Source: Front Oncol. 2020 Mar 27;10:283. doi: 10.3389/fonc.2020.00283 (PMC7119101; doi:10.3389/fonc.2020.00283)
Supplement: Supplementary file 1 [file Table_1.DOC]

**Supplementary Table 1.** Patient information

| **Patient** | **Gender** | **Age** | | **Stage** | | **Grade** | **Metastasis** | |
| --- | --- | --- | --- | --- | --- | --- | --- | --- |
| 1 | F | | 69 | T1 | High | | | N |
| 2 | M | | 60 | T1 | High | | | N |
| 3 | M | | 51 | T1 | Low | | | N |
| 4 | M | | 66 | T1 | High | | | N |
| 5 | F | | 83 | T1 | High | | | N |
| 6 | M | | 77 | T1 | Low | | | N |
| 7 | M | | 81 | T1 | Low | | | N |
| 8 | F | | 62 | T1 | Low | | | N |
| 9 | M | | 59 | T1 | High | | | Y |
| 10 | M | | 74 | T1 | Low | | | N |
| 11 | M | | 60 | T1 | Low | | | N |
| 12 | M | | 82 | T1 | Low | | | N |
| 13 | M | | 59 | T1 | High | | | N |
| 14 | M | | 77 | T1 | High | | | N |
| 15 | M | | 82 | T1 | High | | | N |
| 16 | M | | 78 | T1 | Low | | | N |
| 17 | M | | 82 | T1 | Low | | | N |
| 18 | M | | 63 | T1 | Low | | | N |
| 19 | M | | 79 | T1 | High | | | N |
| 20 | M | | 74 | T1 | High | | | N |
| 21 | M | | 89 | T2 | High | | | N |
| 22 | M | | 84 | T2 | High | | | N |
| 23 | M | | 80 | T2 | High | | | N |
| 24 | M | | 73 | T2 | High | | | Y |
| 25 | M | | 56 | T2 | High | | | N |
| 26 | M | | 56 | T2 | High | | | N |
| 27 | M | | 88 | T2 | High | | | N |
| 28 | M | | 85 | T2 | High | | | N |
| 29 | M | | 70 | T3 | High | | | Y |
| 30 | M | | 68 | T3 | High | | | Y |
| 31 | M | | 84 | T3 | High | | | N |
| 32 | M | | 79 | T4 | High | | | Y |
| 33 | M | | 69 | T4 | High | | | Y |
| 34 | M | | 83 | T4 | High | | | Y |
| 35 | M | | 76 | T4 | High | | | Y |
| 36 | M | | 75 | T4 | High | | | Y |
| 37 | M | | 86 | T4 | High | | | Y |
| 38 | M | | 82 | T4 | High | | | Y |

F: female; M: male. Y: yes; N: no
