# Supplementary material for: Ephrin-B1 Is a Novel Biomarker of Bladder Cancer Aggressiveness. Studies in Murine Models and in Human Samples
Source: Front Oncol. 2020 Mar 27;10:283. doi: 10.3389/fonc.2020.00283 (PMC7119101; doi:10.3389/fonc.2020.00283)
Supplement: Supplementary file 2 [file Table_2.DOC]

**Supplementary Table 2.** PCR primers

A.

| **Murine**  **Primers** | **PCR fragment size (bp)** | **Forward** | **Reverse** |
| --- | --- | --- | --- |
| β-catenin | 113 | CACAGCTCCCCTGACAGAG | GCCGCTTCTTGTAATCCTG |
| E-cadherin | 100 | GCTCTCATCATCGCCACAG | CTGGGATGGGAGCGTTGTC |
| N-cadherin | 79 | GCCATCATCGCTATCCTTC | CCCGCCGTTTCATCCATCC |
| P-cadherin | 151 | CGGGAACTTCATCATCGAGAACCTG | CTGATCCTGGTCGGAGGCGG |
| Ephrin-B1 | 160 | TGGTCGTGCTGACGCTGTGC | GCTTCTGCTCGGGGGCAGATG |
| FZD8 | 122 | GTTCAGTCATCAAGCAGCAAGGAG | AAGGCAGGCGACAACGACG |
| LRP5 | 136 | AAGCAACAGTGTGACTCCTTC | GGGAGAGGATGATACCAATGAC |
| LRP6 | 124 | GGTCTCACCATCGACTATGC | AAGGATGAGGCAAGTCATCTG |
| Snail | 111 | CACGCTGCCTTGTGTCTG | CAGTGGGTGCAGGAGAATGG |
| Slug | 82 | ACATTAGAACTCACACTGGGGA | ACATTAGAACTCACACTGGGGA |
| Twist | 136 | GCTATGTGGCCCACGAGC | GAAACAATGACATCTAGGTCTCCG |
| uPA | 148 | ATCCAGTCCTTGCGTGTCT | AAGTACACTGCCACCTTCA |
| uPAR | 104 | CAGAGCACAGAAAGGAGCTTGA | TGAAAGGTCTGGTTGCTATGGA |
| Wnt3A | 137 | CTGAGCGACGGAGGGAGAAATG | CTCGGAATGAACCCTGCTCC |
| Wnt5A | 122 | AATAACCCTGTTCAGATGTCA | TACTGCATGTGGTCCTGATA |
| Zeb1 | 172 | ACCTGCTGTCGTTCTTTGGAT | AGGGCTTCCTCAGGGAATTT |
| β-actin | 88 | TGCACCACCAACTGCTTAGC | GGCATGGACTGTGGTCATGAG |
| β-actin | 88 | TGCACCACCAACTGCTTAGC | GGCATGGACTGTGGTCATGAG |

B.

| **Human Primers** | **PCR fragment size (bp)** | **Forward** | **Reverse** |
| --- | --- | --- | --- |
| Ephrin-B1 | 94 | GCAAGGAGGCAGACAACACT | TTCACAGTCTCATGCTTGCCA |
| GAPDH | 88 | TGCACCACCAACTGCTTAGC | GGCATGGACTGTGGTCATGAG |
