# Supplementary material for: Ephrin-B1 Is a Novel Biomarker of Bladder Cancer Aggressiveness. Studies in Murine Models and in Human Samples
Source: Front Oncol. 2020 Mar 27;10:283. doi: 10.3389/fonc.2020.00283 (PMC7119101; doi:10.3389/fonc.2020.00283)
Supplement: Supplementary file 3 [file Table_3.DOC]

**Supplementary Table 3**. Gene-disease association analysis in BC samples using DisGENet

| **Gene** | **Association type** | **Score** |
| --- | --- | --- |
| ***CDH1*** | **Biomarker** | **0.6** |
| ***CDKN2A*** | **Biomarker** | **0.6** |
| ***ERCC2*** | **Biomarker** | **0.6** |
| ***FGFR3*** | **Biomarker** | **0.6** |
| ***GSTP1*** | **Biomarker** | **0.6** |
| ***HRAS*** | **Biomarker** | **0.6** |
| ***NQO1*** | **Biomarker** | **0.6** |
| ***TP53*** | **Biomarker** | **0.6** |
| ***TSC1*** | **Causal Mutation** | **0.6** |
| *RB1* | Biomarker | 0.54 |
| *STAG2* | Biomarker | 0.54 |
| *KDM6A* | CausalMutation | 0.53 |
| *CDKN1A* | Biomarker | 0.4 |
| *CXCL8* | Biomarker | 0.4 |
| *EGFR* | Biomarker | 0.4 |
| *GSTM1* | Biomarker | 0.4 |
| *MTHFR* | Biomarker | 0.4 |
| *MYC* | Biomarker | 0.4 |
| *NAT1* | Biomarker | 0.4 |
| *NAT2* | Biomarker | 0.4 |
| *PSCA* | Biomarker | 0.4 |
| *PTGS2* | Biomarker | 0.4 |
| *RASSF1* | Biomarker | 0.4 |
| *TERT* | Biomarker | 0.4 |
| *TNF* | Therapeutic | 0.4 |
| *GPX1* | Biomarker | 0.39 |
| *IL2* | Therapeutic | 0.37 |
| *SOD2* | Biomarker | 0.37 |
| *FAS* | Biomarker | 0.35 |
| *GLI1* | Biomarker | 0.35 |
| *IFNA2* | Therapeutic | 0.35 |
| *KLF5* | Biomarker | 0.35 |
| *ESR2* | Biomarker | 0.34 |
| *IGF1* | Biomarker | 0.34 |
| *IGFBP3* | Biomarker | 0.34 |
| *KRAS* | Biomarker | 0.34 |
| *MTOR* | Causal Mutation | 0.34 |
| *TYMP* | Biomarker | 0.34 |
| *CCNE1* | Biomarker | 0.33 |
| *CSF3* | Therapeutic | 0.33 |
| *POLB* | Biomarker | 0.33 |
| *PRSS3* | Biomarker | 0.33 |
| *TACC3* | Biomarker | 0.33 |
| *UGT2B7* | Biomarker | 0.33 |
| *CYP4B1* | Biomarker | 0.32 |
| *ERBB3* | Causal Mutation | 0.32 |
| *ERCC4* | Biomarker | 0.32 |
| *ESR1* | Biomarker | 0.32 |
| *GSTZ1* | Biomarker | 0.32 |
| *LOXL4* | Biomarker | 0.32 |
| *MPO* | Biomarker | 0.32 |
