# Supplementary material for: Ephrin-B1 Is a Novel Biomarker of Bladder Cancer Aggressiveness. Studies in Murine Models and in Human Samples
Source: Front Oncol. 2020 Mar 27;10:283. doi: 10.3389/fonc.2020.00283 (PMC7119101; doi:10.3389/fonc.2020.00283)
Supplement: Supplementary file 4 [file Table_4.DOC]

**Supplementary Table 4. *CTNNB1* somatic mutations and BC. A bioinformatics analysis using COSMIC**

| |  | **Transcript** | **Sample ID** | | | **AA Mutation** | | **CDS Mutation** |  | | --- | --- | --- | --- | --- | --- | --- | --- | --- | | **ENST00000645320.1** | | | 1511930 | [p.D32Y](https://cancer.sanger.ac.uk/cosmic/mutation/overview?id=150913673) | | [c.94G>T](https://cancer.sanger.ac.uk/cosmic/mutation/overview?id=150913673) | | | | **ENST00000645320.1** | | | 873334 | [p.D32G](https://cancer.sanger.ac.uk/cosmic/mutation/overview?id=150913593) | | [c.95A>G](https://cancer.sanger.ac.uk/cosmic/mutation/overview?id=150913593) | | | | **ENST00000645320.1** | | | [873333](https://cancer.sanger.ac.uk/cosmic/sample/overview?id=873333) | [p.S33F](https://cancer.sanger.ac.uk/cosmic/mutation/overview?id=150913739) | | [c.98C>T](https://cancer.sanger.ac.uk/cosmic/mutation/overview?id=150913739) | | | | **ENST00000645320.1** | | | 2724900 | [p.S33F](https://cancer.sanger.ac.uk/cosmic/mutation/overview?id=150913739) | | [c.98C>T](https://cancer.sanger.ac.uk/cosmic/mutation/overview?id=150913739) | | | | **ENST00000645320.1** | | | 2722156 | [p.S33C](https://cancer.sanger.ac.uk/cosmic/mutation/overview?id=150913524) | | [c.98C>G](https://cancer.sanger.ac.uk/cosmic/mutation/overview?id=150913524) | | | | **ENST00000645320.1** | | | 2721016 | [p.S33C](https://cancer.sanger.ac.uk/cosmic/mutation/overview?id=150913524) | | [c.98C>G](https://cancer.sanger.ac.uk/cosmic/mutation/overview?id=150913524) | | | | **ENST00000645320.1** | | | 2097325 | [p.S37F](https://cancer.sanger.ac.uk/cosmic/mutation/overview?id=150913532) | | [c.110C>T](https://cancer.sanger.ac.uk/cosmic/mutation/overview?id=150913532) | | | | **ENST00000645320.1** | | | 2723306 | [p.S37C](https://cancer.sanger.ac.uk/cosmic/mutation/overview?id=150913731) | | [c.110C>G](https://cancer.sanger.ac.uk/cosmic/mutation/overview?id=150913731) | | | | **ENST00000645320.1** | | | 2723307 | [p.S37C](https://cancer.sanger.ac.uk/cosmic/mutation/overview?id=150913731) | | [c.110C>G](https://cancer.sanger.ac.uk/cosmic/mutation/overview?id=150913731) | | | | **ENST00000645320.1** | | | [2719233](https://cancer.sanger.ac.uk/cosmic/sample/overview?id=2719233) | [p.S37F](https://cancer.sanger.ac.uk/cosmic/mutation/overview?id=150913532) | | [c.110C>T](https://cancer.sanger.ac.uk/cosmic/mutation/overview?id=150913532) | | | | **ENST00000645320.1** | | | 1512039 | [p.S37C](https://cancer.sanger.ac.uk/cosmic/mutation/overview?id=150913731) | | [c.110C>G](https://cancer.sanger.ac.uk/cosmic/mutation/overview?id=150913731) | | | | **ENST00000645320.1** | | | 1512012 | [p.S37F](https://cancer.sanger.ac.uk/cosmic/mutation/overview?id=150913532) | | [c.110C>T](https://cancer.sanger.ac.uk/cosmic/mutation/overview?id=150913532) | | | | **ENST00000645320.1** | | | 873332 | [p.S37C](https://cancer.sanger.ac.uk/cosmic/mutation/overview?id=150913731) | | [c.110C>G](https://cancer.sanger.ac.uk/cosmic/mutation/overview?id=150913731) | | | | [**ENST00000645320.1**](http://www.ensembl.org/Homo_sapiens/geneview?gene=ENST00000645320.1) | | | 870460 | [p.S37C](https://cancer.sanger.ac.uk/cosmic/mutation/overview?id=150913731) | | [c.110C>G](https://cancer.sanger.ac.uk/cosmic/mutation/overview?id=150913731) | | | |
| --- | --- | --- | --- | --- | --- | --- | --- | --- | --- | --- | --- | --- | --- | --- | --- | --- | --- | --- | --- | --- | --- | --- | --- | --- | --- | --- | --- | --- | --- | --- | --- | --- | --- | --- | --- | --- | --- | --- | --- | --- | --- | --- | --- | --- | --- | --- | --- | --- | --- | --- | --- | --- | --- | --- | --- | --- | --- | --- | --- | --- | --- | --- | --- | --- | --- | --- | --- | --- | --- | --- | --- | --- | --- | --- | --- | --- | --- | --- | --- | --- | --- | --- | --- | --- | --- | --- | --- | --- | --- | --- | --- | --- | --- | --- | --- | --- | --- | --- | --- | --- | --- | --- | --- | --- | --- | --- | --- | --- | --- | --- | --- | --- | --- | --- | --- | --- | --- | --- | --- | --- | --- | --- | --- | --- | --- | --- | --- | --- | --- | --- | --- | --- | --- | --- | --- |

Data retrieved from the COSMIC database (GRCh38 · COSMIC v90).
