# Supplementary material for: Ephrin-B1 Is a Novel Biomarker of Bladder Cancer Aggressiveness. Studies in Murine Models and in Human Samples
Source: Front Oncol. 2020 Mar 27;10:283. doi: 10.3389/fonc.2020.00283 (PMC7119101; doi:10.3389/fonc.2020.00283)
Supplement: Supplementary file 5 [file Table_5.DOC]

**Supplementary Table 5.** Immunodetection of E-cadherin in BC samples and association with clinicopathological parameters

|  | **n** | **E-cadherin normal**  **(%)** | **E-cadherin abnormal**  **(%)** | ***P*** |
| --- | --- | --- | --- | --- |
| **Stage** |  |  |  |  |
| T1 | 20 | 12 (60.0) | 8 (40.0) | 0.193 |
| T2-T4 | 18 | 7 (39.0) | 11 (61.0) |  |
| **Grade** |  |  |  |  |
| Low | 10 | 6 (60.0) | 4 (40.0) | 0.463 |
| High | 28 | 13 (46.0) | 15 (54.0) |  |
| **Metastasis** |  |  |  |  |
| No | 27 | 15 (56.0) | 12 (44.0) | 0.283 |
| Yes | 11 | 4 (36.0) | 7 (6.0) |  |
